# What needs to happen for school autonomy to be mobilised to create more equitable public schools and systems of education?

**DOI:** 10.1007/s13384-022-00573-w

**Published:** 2022-09-30

**Authors:** Amanda Keddie, Katrina MacDonald, Jill Blackmore, Ruth Boyask, Scott Fitzgerald, Mihajla Gavin, Amanda McKay, David Hursh, Susan McGrath-Champ, Jorunn Møller, John O’Neill, Karolina Parding, Maija Salokangas, Craig Skerritt, Meghan Stacey, Pat Thomson, Andrew Wilkins, Rachel Wilson, Cathy Wylie, Ee-Seul Yoon

**Affiliations:** 1 Deakin University, Geelong, Australia; 2 Auckland University of Technology, Aotearoa, New Zealand; 3 Curtin University, Bentley, Australia; 4 University of Technology Sydney, Sydney, Australia; 5 Manchester Institute of Education, University of Manchester, Manchester, UK; 6 University of Rochester, Rochester, USA; 7 University of Sydney, Sydney, Australia; 8 University of Oslo, Oslo, Norway; 9 Massey University, Aotearoa, New Zealand; 10 Luleå University of Technology, Luleå, Sweden; 11 Maynooth University, Maynooth, Ireland; 12 University of New South Wales, Sydney, Australia; 13 University of Nottingham, Nottingham, UK; 14 Goldsmiths, University of London, London, UK; 15 New Zealand Council for Educational Research, Aotearoa, New Zealand; 16 University of Manitoba, Winnipeg, Canada

**Keywords:** School autonomy reform, Social justice, Public schooling, Teacher autonomy, Principal autonomy

## Abstract

The series of responses in this article were gathered as part of an online mini conference held in September 2021 that sought to explore different ideas and articulations of school autonomy reform across the world (Australia, Canada, England, Ireland, the USA, Norway, Sweden and New Zealand). It centred upon an important question: what needs to happen for school autonomy to be mobilised to create more equitable public schools and systems of education? There was consensus across the group that school autonomy reform creates further inequities at school and system levels when driven by the logics of marketisation, competition, economic efficiency and public accountability. Against the backdrop of these themes, the conference generated discussion and debate where provocations and points of agreement and disagreement about issues of social justice and the mobilisation of school autonomy reform were raised. As an important output of this discussion, we asked participants to write a short response to the guiding conference question. The following are these responses which range from philosophical considerations, systems and governance perspectives, national particularities and teacher and principal perspectives.

## Introduction

### Amanda Keddie and Katrina MacDonald

The series of responses in this article were gathered as part of an online mini conference held in September 2021 that sought to explore different ideas and articulations of school autonomy reform across the world. The conference was hosted by Deakin University and engaged with over 30 scholars from Australia, Canada, England, Ireland, New Zealand, Norway, Sweden and the United States. It centred upon an important question: *what needs to happen for school autonomy to be mobilised to create more equitable public schools and systems of education?*

The format of the conference was designed to foster comprehensive and critical discussion. As preparation, it involved participants sharing with us a research piece/artifact that spoke to the question (above). We received over 28 papers, blog entries and videos that were shared prior to the conference amongst the group. We began the conference with a presentation of the key issues raised in these artifacts concerning how school autonomy is currently being mobilised across the world within different nation states in ways that undermine social justice.

Consistent with decades of research (see Blackmore et al., 1996; Smyth, 2011) there was consensus across the group that school autonomy reform creates further inequities at school and system levels when driven by the logics of marketisation, competition, economic efficiency and public accountability (e.g., these logics force public schools to run themselves like businesses, prioritise narrow outputs and compete for students which leads to stratification and residualisation within education systems and inequitable resource allocation for students) (Constantinides, 2021; Fitzgerald et al., 2018; Karseth & Møller, 2020; Keddie et al., 2020a, 2020b; Lundahl, 2019; Wilkins et al., under review). Consistent with ongoing concerns in this space, there was a questioning of the idea of the public in public (Gerrard, 2018) and private education (Boyask, 2020, 2021) in terms of who and how education operates for the public good and relatedly; about how private sector interests and logics have permeated public school governance to prioritise market imperatives at the expense of educative imperatives (Hursh, 2017; Lipman, 2017; Lubienski, 2009; O'Neill, 2021; Skerritt, 2019; Skerritt & Salokangas, 2020; Thrupp, 2020; Yoon et al., 2020); about how the increased expectations and responsibilities associated with school autonomy reform were continuing to take an enormous physical and mental toll on teachers and school leaders in relation to untenable work intensification (Fitzgerald et al., 2018; McKay & Pierpoint, 2020; Keddie et al., 2020a, 2020b; Skerritt, 2020; Wilkins et al., under review; Wylie, 2020); and about how these reforms as they are driven by a narrow performative culture, continue to degrade pedagogy and curriculum towards a teach-to-the-test mentality (Hursh et al., 2019; McGrath-Champ et al., 2018). There were also concerns raised about the new articulations of the governing parent-citizen within the context of education devolution as reconfiguring what a ‘good’ parent-citizen looks like along strongly classed and professionalised lines (Gerrard & Savage, 2021) and finally, concerns were raised about how union power might be mobilised to resist the processes of devolutionary reform (Gavin et al., 2022).

Against the backdrop of these themes, the conference generated discussion and debate where provocations and points of agreement and disagreement about issues of social justice and the mobilisation of school autonomy reform were raised. As an important output of this discussion, we asked participants to write a short response (500–750 words) to the question: *What needs to happen for school autonomy to be mobilised to create more equitable public schools and systems of education?* The following are these responses which range from philosophical considerations of the question, systems and governance perspectives, national particularities and teacher and principal perspectives.

## School autonomy: fictions of empowerment or vehicles for self-actualisation?

### Andrew Wilkins

The concept of autonomy has a special resonance for people living in social democracies or societies that are transitioning to social democracies. Derived from the ancient Greek words autos, meaning ‘self’, and nomos, meaning ‘rule’, and later popularised in the eighteenth and nineteenth centuries by the liberal philosophies of Immanuel Kant and John Stuart Mill, autonomy conjures up some very seductive fantasies. This includes the “individualist fiction of the disembodied or unsituated human subject” (Gray, 2007, p. 24), namely the classical liberal idea of the sovereign, self-originating subject, or what is sometimes called the ‘Cartesian subject’. Related to this is a distrust of authorities, such as the church or the state, which are thought to constrain the self-regulating development of subjects.

Similarly, the concept of school autonomy, with its emphasis on publicly funded schools operating ‘independent’ of traditional structures of government, echoes and redeems a romantic, utopian notion of the self-directed agent. A rhetorical move made popular by “reluctant state[s]” (Ball, 2012, p. 89), namely national governments that have chosen to abdicate responsibility for any direct monitoring or management of their public services, has been to bolster an image of the self-regulating, autonomous school through promoting a dichotomy of “the people versus the state, with the people requiring rescue from an over-bearing, intrusive and dominating public power” (Clarke, 2005, p. 449). Through this people-state dichotomy, key political and bureaucratic authorities, including trade unions and local governments, are characterised as impediments to the ‘innate’ capacity of individuals and organisations to innovate, experiment and self-govern.

Under reforms to make English schools ‘autonomous’, school leaders and governors are delegated discretionary powers to make decisions about strategic planning and budget spending. In effect, local government agents (elected councillors, civil servants) are supplanted in their role as knowledge brokers, admission officers, dispute handlers, and risk controllers, with the bulk of these responsibilities devolved to school leaders and governors as site managers. While school autonomy as a global policy movement is uneven owing to the unique path dependencies of different countries, the rhetoric is a universal one: school autonomy improves school efficiency and effectiveness through displacing the slow, cumbersome bureaucracy that characterises traditional public administration. It is precisely this rhetoric that makes school autonomy such a powerful fantasy object for school leaders and governors who attribute much of their dissatisfaction to the role of authorities, be it an intractable bureaucracy or an overbearing state.

Yet the reality is not a post-bureaucratic one. Schools with ‘autonomy’ still operate within the shadow of the state as their autonomy is made conditional on them inhabiting and performing new kinds of responsibilities and obligations, including the role of purveyors of utility, market logic and business ontology (see Gobby & Niesche, 2019; Hetherington & Forrester, 2021). The model of equity made possible by democratically controlled forms of public education has never been ideal owing to geographical disparities in local government efficiency. But the reality of unwieldy democracy seems far preferable to the fantasy of school autonomy which now prevails.

## Rupturing the political power of the autonomous medieval commune in 21st century school governance

### Ruth Boyask

Walking through the fortified grounds and medieval, Tudor, and Gothic architecture there is a visual resemblance between United World College of the Atlantic (UWC Atlantic) and the ideal democratic city-states of Europe. The privately financed senior secondary school is located on the coast of South Wales in the splendid St Donat’s Castle. UWC Atlantic also appears to stand ethically like the medieval communes against rule by unitary or state authority and engages students in the development of their own political will. The school, “focusses on a lifelong commitment to service in the community, to collaborative work and social engagement and develops in young people a sense of personal initiative and leadership” (UWC Atlantic, n.d.).

Max Weber’s (1921/1958) reflection on *The City* is an analysis of the formation of modern democracy (Gianola, 2021), centred upon the medieval commune and its peculiar characteristics. The commune wrested its democratic power through struggle and usurpation of feudal power, or a rupture in the legitimate order. Weber’s argument was that 20^th^ century democracy was closer to what was founded in medieval communes than Athenian democracy. The power of the administration within the commune to legislate (autonomy) and rule itself (autocephaly) was tied to commercial enterprise, with councils made up of merchant representatives and their governance work centred on interactions with guilds (the main producers) or legislating property rights, forming a capitalist economic system (Stasavage, 2014).

In the present-day, UWC Atlantic has carved out its niche within the political and economic establishment. It is known as a school attractive to royalty (Vanderhoof, 2021). Its capacity for self-rule is permitted not through rupture but sustenance of the existing order. This is the case for many privately funded schools, where innovations in democratic governance and participation are severely curtailed and even if present are generally restricted to within-school relations (Boyask, 2020).

In public sector schooling, origins of school autonomy are found in economic-orientated education reforms in the late twentieth century, such as the Tomorrow’s Schools (1989) reforms of New Zealand with their shift from centralised administration to independent school boards, or in Sweden from the early 1990s a system of public voucher funding used to establish an industry of “free schools” (Lundahl et al., 2013). These experiments in school governance took root in the twenty-first century, with parallel policies in America’s charter schools, independent public schools in Australia and England’s Academies program. These schools resemble more fully than Atlantic College the medieval communes in that their political power is put to work on regulation of students’ (the expanding demos) interests in the twenty-first century economy. They do not cater just to an elite. Yet in these schools, freedom is predominantly the freedom to participate in an existing economy, and only weakly associated with broader concepts of justice (Boyask, 2020).

Using the term autonomy to describe ideals of democratic freedom and justice in education is difficult because it brings with it confounding connotations (Olssen, 2005). In answer to the question guiding our responses, values of freedom and justice associated with autonomy are worth retaining in education but retaining them while educating for a Eurocentric capitalist economy compromises their quality. A system of education with autonomous schools reproduces existing social relations of capital. In education we might get closer to an education of justice through release from the history of the autonomous city-state and rupturing aspirations for school autonomy.

## Creating more equitable public schools and systems of education

### Jorunn Møller

For many years, the OECD has effectively advocated for school autonomy as a desirable form of educational governance to increase quality in education and to address achievement gaps across cultural groups (OECD, 2012; Schleicher, 2014). The OECD’s recommendations for more school autonomy have implied a strong push to develop accountability instruments with a clearly pro-market purpose. The seductive power of these accountability instruments is related to the way they tend to transform complex educational realities into numerical categories (Verger et al., 2019).

The problem with measurable outcomes is that it drives distortions of curriculum as a result of benchmarking and teaching to the test. It also increases the game-playing strategies of those with most cultural capital to maintain their position. Even when the national government does not publish school scores, (as in Norway) many local governments or media do so, taking advantage of transparency rules in public administration (Camphuijsen & Levatino, 2021), and the discourse of quality in education is reduced to measurable outcomes. In addition, processes of juridification in education indicate a more detailed legal regulation and a tendency to frame emerging problems or conflicts in legal terms (Karseth & Møller, 2020). It has led to increased individualism and the idea of education as a private good.

Studies based in Anglo-Saxon countries, which have highlighted stories of far-reaching marketisation and privatisation of education might serve as a cautionary tale to learn from. It is necessary to stand up against politicians who argue that marketisation reforms can mobilise teachers and school principals to do better than before, because there is an uneasy tension between public and private good embedded in such arguments. It is difficult to see how a mixed public/private education system relying on a possessive individualism could prepare citizens better for the communicative society than a public education system that provides the right of the child to encounter the pluralist society within the school (Møller & Rönnberg, 2021).

Key questions to ask are whether the role of education is to provide a public good, a good for the country as a whole, or is it an individual private good? Which conditions will sustain education as a public good? First, a warning: we should not engage in a romantic retrospect for good old public education that we may have lost or are about to lose. We know that public education in the past too often was incapable of effectively promoting practices that were ethically sound (Anderson & López, 2017). In shaping more equitable public schools and systems of education it is crucial to understand the larger social and policy environment in which teachers and principals work and develop collective strategies to limit their failing effects on educational inequalities (Takayama, 2013).

Although we should never underestimate the power of schools and educators to influence students’ learning and learning environment, the out-of-school factors associated with poverty or racism will always play a powerful role and limit the role of creating equitable public schools (Møller, 2017). Without social and economic reforms to accompany reforms in schooling, issues of inequity will persist in the future.

## Moving from morally improper to morally proper school autonomy

### Craig Skerritt

It may be easier to begin by focussing on how school autonomy should *not* be mobilised. That is, it should not be used in ways that: impose strict accountability regimes; prioritise external measures; force competition; and detract from broad and meaningful experiences of teaching and learning and compromise teachers and learners, increasing the likelihood of engendering inequalities. I write here with specific reference to England's academy schools. Teachers frequently paint damning pictures of academies and although the voices of students are often underrepresented in this literature, it would be naive to think that their views are too dissimilar. A recent small-scale study by Lewis and Pearce (2021) highlights how caring relationships between students and teachers are neglected due to examination success and positioning in the education marketplace being prioritised. As this autonomy rises so does accountability which produces highly pressurised environments. Thus, academies’ autonomy might be thought of as “coercive” (Greany & Higham, 2018) or “indentured” (Thompson et al., 2020). We can to some degree sympathise with the leaders of these quasi-autonomous schools but we should be mindful of the many reports of autocratic and authoritarian academy leaders. Writing about toxic leadership, Ian Craig (2017) warns that the academy movement could exacerbate the trend of school leaders becoming more directive at the expense of teachers’ professional autonomy.

The above has had me think of some interesting posits of critical scholars: despite the widespread perception that schooling is a good thing, it is too simplistic to say that it has indisputable benefits (Gewirtz & Cribb, 2009); modern schools are intolerable and irredeemable (Ball & Collet-Sabe, 2021); and ‘ordinary’ schools no longer exist in England (Maguire et al., 2011). School autonomy is, theoretically, difficult to argue against. This freedom could, for example, be in many ways redeemable. School autonomy should be mobilised in ‘morally’ ‘proper’ ways such as focussing on students and their learning instead of external tests and measures (Keddie, 2014). Indeed, teachers I previously interviewed saw merit in this autonomy if it could be embraced and enacted in morally focussed ways as opposed to focussing on extrinsic motivations (Skerritt, 2019) but the current context in which academies operate largely determines otherwise. It is not the micro-level we need to zoom in on here but the macro. Schools all have their own contexts but when they operate in a wider marketised, competitive, accountability-driven one, how can we expect them to act in morally proper ways?

We need to go back to the drawing board and eradicate the hegemonic culture that has become normalised and that too many have been socialised into—these issues go far beyond academies but with their freedom comes responsibility, and this responsibility is too easily displaced by contemporary accountability regimes. One possibility for mobilising autonomy in morally proper ways could be to move from typical external assessments to concentrating on how schools promote teachers as professionals. Although not writing about school autonomy, Sharon Gewirtz and Alan Cribb (2020) provide good food for thought. These authors draw on ideas from the Netherlands that propose schools be required to demonstrate how professionals are enabled to apply and enhance “their professional expertise and experience, to exercise their professional discretion and to remain dedicated to the values of their profession” (Gewirtz & Cribb, 2020, p. 228). Thus, schools driven by, for example, market success would not perform well here (Gewirtz & Cribb, 2020). This could be one way of reducing the risk of academies acting in morally improper ways.

In a recent paper, I write about questioning contemporary accountability, which should require parents and the public to come together with teachers (Skerritt, 2022). As widespread opposition has previously prevented a full-scale academisation of English schools, social action could help to mobilise school autonomy in moral ways. Perhaps the way forward for school autonomy lies in communities’ hands—as we know too well from the metrics that dominate English education, there is strength in numbers!

## School autonomy, fundraising inequities, and neoliberal governance

### Ee-Seul Yoon

The rise of neoliberal governance has steadily replaced the public sector with market-based mechanisms whereby individuals find their own solutions while taking on greater financial responsibilities (e.g. paying user fees) with the supposed benefit of paying lower taxes (Klein, 2007). In education, devolution within the public system has ushered in greater school autonomy, especially over financial matters, including school fees and fundraising. Public school administrators and parents are increasingly encouraged to raise, with limited oversight, private sources of funding for educational activities, and to do so as a way to make up for the reduction in government funds (Winton, 2018). In Canada, most schools have nearly complete control over how much money they can raise, as well as how they raise and spend it. Such decisions are made almost exclusively by the school’s Parent Advisory Council (PAC) and school administrators. Fundraising activities and outcomes have led to growing resource inequities between the ‘have’ and ‘have-not’ schools according to the economic and social capital of the school community members who can contribute to fundraising efforts (Yoon et al., 2020). As a result, school autonomy over fundraising is making public school systems less equitable, as schools increasingly rely on their own solutions to funding inadequacies, which reproduce and co-produce the structural inequalities of the broader society.

For instance, in the increasingly unequal global city of Toronto, which is also one of the most diverse cities in Canada, fundraising differs vastly among schools. According to the 2018 People for Education Annual Survey report (https://peopleforeducation.ca/report/fundraising-and-fees-in-ontarios-schools/), “the top 10% of fundraising elementary schools raised 37 times the amount raised by the bottom 10%”. The schools in the top group raised over CAN$100,000. Among secondary schools, “the top 5% of fundraising schools raised as much as the bottom 81% combined”, while the top schools raised over CAN$150,000. Autonomy over how to spend these funds is left to administrators and parent groups, including paying for school library books, art supplies, sports equipment and activities, guest speakers, field trips, and other special events for engaging students with exciting learning opportunities. Spending on these enriched learning opportunities is in itself not a problem, but becomes a societal issue if these opportunities are not available to all children, especially those who start their educations in a disadvantaged social position.

This type of school autonomy, especially with respect to fundraising, should thus be regulated, if not eliminated entirely, because it allows affluent families to mobilise resources for high-quality education only for their children. They do so, moreover, while withdrawing from supporting a system-wide change in funding that is critical in ensuring that all schools, especially schools in high-poverty areas, get the resources they need. It is paramount, in an increasingly diverse yet unequal world, including Canada, that we demand that our school systems value and improve equity for students who come from families that are disadvantaged due to poverty, unemployment, migration, displacement, and other challenges. Efforts towards more equitable resource distribution can start with retracting neoliberal governance; that is, to stop seeking policy solutions that continue to benefit those who are already advantaged.

## A view from the USA

### David Hursh

Over the last 30 years, New Yorkers have been involved in a struggle over public education. Some, like the authors of *A Nation at Risk* (The National Commission on Excellence in Education, 1983) and other proponents of privatising education, assert that public schools are failing because teachers, students, and parents are not working as hard as they can. Therefore, teachers cannot be autonomous but need to be held accountable through standards and standardised tests that provide objective measures of what students are learning.

In contrast, the 38 public K-12 schools that comprise New York’s performance assessment consortium assess students not on standardised tests but on whether students have demonstrated that they have achieved proficiency in a lengthy list of capabilities. The Consortium schools state that they have developed a performance assessment system that is “practitioner-developed, student-focused, and externally assessed” (http://www.performanceassessment.org/memberschools).

Here, I maintain that the last 30 years of neoliberal reforms focussing on quantitative assessment, privatisation, and top-down decision-making neither increased student learning nor closed the so-called ‘achievement gap’. Rather, they have worsened educational outcomes, for which there are three general reasons. First, neoliberal reformers pay little attention to the macro causes of student failure: child poverty, significantly unequal school funding, and inadequate housing, childcare, and healthcare. Second, the push to privatise schools through publicly funded privately operated charter schools or diverting public funds to private schools by giving families vouchers is reducing the funding available to public schools. Several state legislatures are currently considering passing bills that would eliminate funding for public schools. Third, standardised testing has not provided more objective assessments but has often intentionally been manipulated by raising or lowering the “cut score” (the score students need to achieve to pass the test or be deemed ‘proficient’), to achieve the politically desired results. For example, during the first administration of the Common Core exams, neoliberal reformers aimed for a 10–15% passing rate and were pleased when so few students passed, because, they claimed, it demonstrated the need for school privatisation.

Currently, not only are neoliberal policies portraying schools as failing, cultural and political conservatives are pushing to remove from schools any books that present anything other than what they think reflects ‘critical race theory’. Consequently, because they are one of the few places that are open to the public and typically anyone at the meetings can speak, school board meetings have become the site of vociferous disagreements over curriculum, and COVID mask mandates.

Schools, then, are at the centre of the vortex. We need to respond to the structural inequalities that contribute to our educational failures. Restoring serious debate on the purpose and nature of education will be difficult, requiring that we develop trust in one another (Sahlberg & Walker, 2021). We will need to develop places where we can begin to create the structures and processes that support trusting relationships. This will be difficult but necessary. Perhaps, schools can become the places where not just children but also adults can learn to become democratic citizens.

## A view from New Zealand

### John O’Neill

In the Aotearoa New Zealand policy vernacular of the late 1980s, a new ‘educational myth’ of autonomy promised to meld: (i) social democratic sentiments of self-governance of the schooling commons by local communities; (ii) market-liberal economics of school choice by consumer nuclear families; and (iii) New Public Management accountability of teachers for the reduction of structural education. Myths such as school autonomy both encourage societal commitment to working towards a shared educational ideal that may never be fully realised, and “make possible a working relationship between people whose attitude towards social change are at odds, and probably always will be at odds” (Beeby, 1986, p. xxvi).

What has eventuated in practice over more than 30 years is not so much a tightly coupled national system but a loosely coupled collection of two and a half thousand self-managing statutory Crown entities. In key respects, school trustees and executive principals as a whole have become distrustful of and resistant to any system-wide intervention that appears to constrain the freedom of each school to act in the self-interest of its particular parent community: identity, needs, and aspirations.

This fracturing of the progressive notion of schooling as inclusive, unitary nation building has been compounded by a shift in the spending priorities of successive governments, from full public funding and provision of schooling, to partial public subsidy in combination with expectations of entrepreneurial activity at the school level (e.g. international student revenue) and facilitation of market entry to contestable schooling services provision for a plethora of commercial, philanthropic and community actors. The national Ministry of Education is now but one node in a complex social network of immanent relations and tactical and strategic alliances that each school navigates in order to maximise its share of the limited “common pool resources” (Ostrom, 2015) offered by the state.

Borrowing Taylor’s (2003) concept of the modern social imaginary, there is now a widespread everyday understanding among lay school trustees and executive principals that these practices are legitimate and therefore unquestionable. They materially reward governance and management initiative, resilience and self-reliance at the local level. This in turn is consistent with a contemporary meritocratic norm that asserts successful schools are successful entirely because of their own independent efforts and achievement, and not because politically and ideologically motivated system settings reinforce their existing social and economic advantages and encourage ‘free-riding’ rather than the altruism essential to improved system equity.

As recent Indigenous iwi Māori experience illustrates, both recognition and redistribution (Fraser & Honneth, 2003) are required if public schools and schooling are to remove the ‘unfreedoms’ that prevent many individuals, families and communities from developing the capabilities to realise their best interests (Nussbaum, 2011). Mutual recognition, attentiveness and solidarity within institutionalised relationships (such as schooling) are essential to the achievement of social freedom (Honneth, 2014); this is a quite different view of the role of autonomy in equitable schooling.

## Social justice and school autonomy reform requires activist- rather than market- oriented public education systems – A view from Australia

### Amanda Keddie

Exploring the social justice implications of school autonomy reform has been the focus of research for decades in Australia and globally. While the expectations of this reform are that greater autonomy (over staffing, resourcing and programs) will allow schools to innovate, better respond to local needs and thus raise academic attainment, the reality is that such autonomy is always subject to the governance mechanisms of the systems within which they operate. For decades, these mechanisms have increasingly been driven by market imperatives of economic efficiency, competition, and external auditing.

These imperatives have re-articulated the priorities, purpose, and performance of schools with clear social justice implications. In particular, they have whittled away systemic support for schools, expecting that schools do more with less, which has led to untenable work intensification for many principals and teachers. The increased external public auditing of education has reduced school improvement to narrow performative outputs and promoted competition between schools. These conditions have increased (classed and racialised) stratification and residualisation within the system advantaging already advantaged schools and disadvantaging already disadvantaged schools (Keddie et al., 2020a, 2020b; Lamb et al., 2015). As many scholars have argued, these conditions and forces have undermined a collective approach to education as a public good. Indeed, they have rendered unstable the political claim of education as a common good (in terms of its deep tradition of democratic participation and active informed citizenship) (see Smyth, 2011; Gerrard, 2018).

Our project is exploring the social justice implications of school autonomy reform in public education systems across four Australian states (see Keddie et al., 2020a, 2020b). We are focussing on understanding how economic, cultural and political justice might be more possible within varying contexts and policies of school autonomy reform (https://www.schoolautonomyandsocialjustice.org). It is unsurprising that our project is generating similar insights about the social justice implications of school autonomy reform as those that have concerned researchers for decades. Given these enduring concerns, how might we respond to the question: *what needs to happen for school autonomy to be mobilised to create more equitable public schools and systems of education*? On reflection, a more apt question would be: *what do public education systems need to do to better support schools to mobilise their autonomy for social justice?* This is a better question because it draws attention to the ongoing significance of re-thinking the system within which school autonomy operates. It leads to re-thinking the market imperatives of economic efficiency, competition and public accountability that move schools away from their public purpose. More than 10 years ago, Smyth (2012) offered three key propositions for a politically activist view of school autonomy which remain as urgent now as they were then. We re-articulate these propositions as questions for education systems.

Towards a politically activist system, we ask, how can systems of public education be reformed so that:

1. schools are not driven by a quest for possessive and competitive individualism within and between themselves but rather a concern for community and collective action?
2. decisions about schooling are informed much more by educational considerations than those of economics (and) entrepreneurialism?
3. schools are engaged in questioning what it is they are doing, not from an accountant’s point of view, but from the perspective of how their agenda fits with a broader view of what constitutes a just society?

Market imperatives currently driving education systems continue to shift school priorities away from their public purpose of creating more inclusive and democratic societies towards private purposes of creating human capital and social mobility (Cranston et al., 2010, p. 520). To rebalance school priorities towards their public purpose so that schools can mobilise their autonomy for social justice requires activist not market-oriented education systems.

## Is school autonomy inherently good?

### Cathy Wylie

My first reaction to the question *What needs to happen for school autonomy to be mobilised to create more equitable public systems and systems of education?* was that it was the wrong way round. After more than 30 years of school autonomy in Aotearoa New Zealand within a laissez-faire system, the abiding question is *What public systems and systems of education are needed to ensure that school autonomy is equitable*? Although improving inequities of access, experience, and outcomes was a prime rationale for this country’s shift to schools operating autonomously, we are proof that school autonomy is not inherently good. The concerns around inequitable student outcomes and variable school performance that fuelled our shift have only become deeper and more urgent to address.

I can see the appeal of school autonomy in other education systems. Where schools have to operate within very bureaucratic regulations, over-determined and ill-fitting accountabilities, with rigid measures of student performance. Where the ability of school leaders to sustain strong and progressive teaching and learning practices that respect and build on the particular strengths and needs of the school’s students is undermined by their not being given sufficient latitude.

I can see that school autonomy seen through a community-serving lens is important. But our experience urges caution here. Our schools are governed by parent-elected boards of trustees, and there are expectations of community consultation. But what that means differs markedly in different contexts, and can contribute to further inequity. Our schools were encouraged to focus on what made them unique. Often this encouraged a marketing perspective: what would make them attractive to students and their families? To desirable students? Over time, too much autonomy over enrolments has increased competition between schools, leading to increased inequities, including white flight and insufficient resourcing for schools serving disadvantaged communities. It has also weakened the sharing and building of effective practice.

Making school autonomy the centrepiece of an educational system did not make individual schools in Aotearoa New Zealand more equitable, because they were insufficiently supported and connected. Our schooling reform occurred in the era of new public management, when separation between policy and operations was deemed essential. That meant cutting out advice and joint work, so over time there was a loss of curriculum expertise and understanding of schools as complex enterprises, as well as insufficient support for school leaders. Variability between schools becomes the hallmark of such a system, as does wearying and costly re-invention of the wheel, without much to show for it in terms of improved equities. That’s why a Labour-led government asked for a fundamental review of our schooling system in 2018, and why it accepted most of this review’s recommendations (Ministry of Education, 2019; Tomorrow’s Schools Independent Taskforce, 2019). These recast the schooling system as an ecosystem in which schools have the customised nourishment they need, while contributing to the wider community of schools and practice around them. Even before COVID-19 delayed the start of this recasting until late 2021, it was clear that it will take some years to rebuild the capability and capacity needed to tackle the inequities that plague Aotearoa New Zealand schooling, and to restore that essential lubricant in any worthwhile education system, trust.

## School autonomy – a view from England

### Pat Thomson

In England, schools have been locally managed since the late 80s. The vast majority of serving school leaders have known nothing else. While concerns about equity, datafication, workload, regulation via inspection and tests and exams and the costs of competition are part and parcel of everyday life and attributed to a system organised around centralised governance and local management (e.g. Courtney, 2015; Gunter & McGinity, 2014; Hutchings & Francis, 2018), head teachers highly value the capacity they have to make local decisions that suit local needs and interests.

However the locally managed school and system is now undergoing radical change. Local authorities have been displaced in favour of academies and multi-academy trusts (MATs).

The majority of secondary education is now academised but the majority of primary education remains with financially struggling and policy-derided local authorities. Pre-pandemic, the government was pushing to fulfil their vision of all schools being academies. They also wanted to move standalone and small trusts into larger MATs more likely to control and support their schools (Ball, 2018; Bernardinelli et al., 2018; Wilkins, 2017).

Trusts have taken various views of what control and support means. Many MATs have removed some of the freedoms that local management gave to local schools. MATs usually:

- have a corporate identity—school websites, and prospectuses indicate the “brand”.
- take a proportion of the budget allocated to each member school. This sum is intended to cover administration, development and support. MATs often vary the amount required of member schools depending on their perceived needs, and they often redistribute funds between schools. Practices related to school budgets also vary – in some instances heads are no longer in charge of their own budgets, staffing and resourcing decisions, while in others there is still the same degree of autonomy as in pre-trust days.

MATs have also understood instructional improvement differently with some demanding all member schools follow a centrally determined program, pedagogy and disciplinary approach. Other MATs have retained and valued local differences and school based curriculum. MATs approach governance differently too, with some removing governing bodies altogether, while other MATs have retained a strong commitment to local consultation and decision-making (Thomson, 2020).

The pandemic brought further systemic changes, increasing centralised control. Emergency Covid19 legislation allowed central government to take over procurement and make national decisions about closure, remote learning and working patterns which ignored local contexts, expertise and needs. Tone-deaf political rhetoric, inefficiency, corruption and flurries of last minute advice together with this loss of school capacity to make key decisions have led to the anger and alienation of leaders of both MATs and local authority schools. Escalating poverty and viral demands on health and welfare services saw many schools become the last public service standing in many communities. In the face of cavalier Westminster interventions, many MATs actively took on a public ethos and commitments, and new networks between MATs and local authority schools were formed, often with the support of the local authority. United in opposition to the failures of central government and committed to their local communities, they kept schools open in the most challenging circumstances (Greany et al., 2021).

Whether these new arrangements hold remains to be seen. But there is at present a renewed interest in the profession in how the English school system might be re-organised and regulated in ways which restore local autonomy and accountability, changing once again the locus of decisions and delegations. The government, however, seems very determined to fully academise the entire school system.

## Teachers’ work and working conditions under school autonomy

### Meghan Stacey, Karolina Parding, Rachel Wilson, Scott Fitzgerald, Susan-McGrath-Champ, and Mihajla Gavin

For school autonomy to create a more equitable school system we need stronger recognition of how teachers are positioned by and implicated within autonomy models. Often, research on school autonomy has focussed on perspectives other than those of teachers, such as principals or students (e.g. Keddie et al., 2020a, 2020b; Parding & Berg-Jansson, 2016). However, the Teachers’ Work project has examined teachers’ work and working conditions in Australia and Sweden over the past decade during the implementation of significant autonomy reforms, and highlighted clear flow-on effects of such reform for teachers. In this brief contribution, we summarise some of the findings of this project to shed light on the work and working conditions of teachers in relation to school autonomy.

In NSW, Australia, two studies explored teachers’ experiences during the implementation of the *Local Schools, Local Decisions* reform, 2012–2020. The first drew on 31 interviews (Stacey et al., 2020), and the second a survey of 18 234 teachers (McGrath-Champ et al., 2018). Both focussed primarily on the issue of teacher work and workload. Teachers were found to perceive their hours to have grown during this period of reform, experiencing heightened administrative demands and increasingly fractured relationships with school leadership. Similar results emerged from our research in WA on the *Independent Public Schools* (IPS) initiative (Fitzgerald et al., 2019). Once again, surveyed teachers reported an expansion and increased administrative complexity of their workload, a pattern of change corroborated by separate surveys conducted by the state teacher union in 2016 and 2021 as IPS became fully established in the public education sector.

In Sweden, we carried out a large-scale stratified survey, via Sweden Statistics, with some 5000 Swedish upper secondary teachers (Parding & Berg-Jansson, 2018), as well as interviews with over 30 teachers and eight principals, in one region, reflecting three different market types. Findings indicate that the school choice reform along with devolution has amplified heterogeneity in conditions for work amongst teachers. Even within a single municipality, factors such as the different financial situations of different schools and contrasting student enrolment patterns have substantial impact on teachers’ conditions for work. We have noted similar patterns in Australia (e.g. Fitzgerald et al., 2018).

That school autonomy can be mobilised to create more equitable public school systems is not a premise we necessarily accept. Where autonomy is focussed on resource management rather than curriculum and assessment (OECD, 2018), as has largely been the case in Australia and Sweden, the potential for equity is constrained. Under such models, resourcing can be limited and student enrolments polarised, as teaching becomes overly focussed on accountability and regulatory reporting rather than core teaching and learning work.

Considering our examination of teachers’ work across these devolved contexts, we argue that teachers have been an important missing voice. Teachers’ experiences of such reform have been largely negative ones. If school autonomy is to be mobilised towards social justice ends, the experiences and views of those who work most closely with students need to be listened to.

## Let’s ask teachers

### Maija Salokangas

In many countries, school autonomy refers to an arrangement in which the school, or rather its management enjoys increased freedom over various aspects of school governance and management, concerning, for example, budget and human resources. In some contexts school autonomy encompasses also educational/pedagogical matters such as freedom from national curriculum. Arguably schools in such systems have increased decision-making capacity over educational/pedagogical matters. However, research shows that in countries with high stakes exams, and external inspection practices, autonomy over curriculum and other educational matters is difficult to utilise as these steering mechanisms dominate pedagogical decisions (Kauko & Salokangas, 2015; Salokangas & Ainscow, 2018). Teachers simply can’t veer from the tight frame such steering imposes.

This points to the role of the teacher and the nature of the teaching profession in an autonomous school. Considering that teachers working in autonomous schools are subject to similar, and sometimes even greater control than their colleagues in other (less-autonomous) publicly funded schools, how autonomous can we really call schools where educators are tightly controlled? Furthermore, if school autonomy reforms are introduced to tackle educational achievement particularly to improve attainment of disadvantaged students, as often is the case, why are the solutions that school autonomy reforms bring to these educational and social problems, in Lubienski’s terms (2003) managerial and/or administrational, rather than educational or social?

For a school to be able to respond to such complex educational problems locally, teachers should be brought into the forefront of reforms and granted decision-making capacity that expands way beyond their current remit, concerning at least the following:

- First, a wide variety of *educational matters* concerning curriculum, assessment, and pedagogy.
- Second, teachers should have a say on *social matters* such as how special needs education is organised. This could be done in conjunction with other professionals such as special needs co-ordinators, school psychologists, and social workers.
- Third, teacher involvement in important *administrative matters* concerning how teaching and learning is organised is central. This does not refer to time-consuming performative red tape, but rather involving teachers in important administrative issues that create conditions for learning in schools such as the distribution of resources, and timetabling.
- Finally, teachers should have a say on *developmental “big picture” matters*, such as: the directions their school is taking and decisions concerning the kind of professional development they engage individually as well as a collective.

Due to the complex and risky nature of educational decisions (Biesta, 2015), such increased decision-making capacity at the local level needs to be paired with appropriate supports, rather than simply control and accountability.

Research tells us, that to be able to solve complex educational problems, teachers need a golden balance of autonomy and supports: such as initial teacher training, professional development, and sufficient resources (Wermke & Salokangas, 2021). What this ideal scope of action for teachers looks like depends on the country context in which the teachers operate (Salokangas & Wermke, 2020; Salokangas et al., 2019), which is why there is no one size fits all solution. However, what could be done in different country contexts is to bring teachers to the forefront of autonomy reforms and ask them what is needed in their schools and classrooms to create a more equitable system of education.

## Placing trust in principals’ expertise at the centre of public education

### Amanda McKay

The current state of autonomy and accountability in Australia’s public schools has largely de-professionalised principals. It increasingly represents a more ‘imagined’ autonomy than ‘real’ autonomy for them to be able to make decisions that meet their community’s needs. There are serious inequities in Australia’s public school principals’ experiences of autonomy. Career stage, length of time in a particular school or district, and the level of advantage in a school community all play an important role in principals’ sense of autonomy (McKay & Pierpoint, 2020; Niesche et al., 2021).

Disadvantaged schools are often further disadvantaged by autonomy policies (Keddie et al., 2020a, 2020b). Principals in these schools tend to be earlier in their careers (Béteille et al., 2012) and less confident in their autonomy to push back against narrowing policy discourses. These principals experience the increased surveillance and steering from a distance that accompany school improvement policies in ways that principals in more advantaged schools do not. Externally imposed targets and measures are felt keenly in already-disadvantaged schools, where principals tend to feel constrained by policy discourses and less able to argue for a different vision of schooling than that valued by reductive school improvement approaches (Keddie, 2017). Moving beyond the rhetoric of autonomy, these external measures are increasingly so specific in Australian schools that they set the agenda for how principals might lead for improved student outcomes. Inequities inherent in these issues of autonomy have consequences for how principals can exercise their expertise, with local priorities being side-lined by externally mandated targets and agendas.

This has implications for principals’ workload and their sense of themselves as leaders. Principals have conveyed frustration at increased compliance and reductions in their ability to lead in ways that address issues of equity and social justice (Gobby et al., 2017). They go into the profession describing wanting to make a difference for young people, but instead feel pressure to meet narrow measures of achievement. They have described feeling micro-managed and cite a lack of systemic trust and respect for their expertise and experience, as well as their local knowledge about their community’s needs. This lack of trust and support has consequences for issues of attraction and retention (McKay & Pierpoint, 2021).

Principals’ expertise and commitment to long-term sustainable improvement needs to be placed at the centre of policy and practice. A shift toward real, rather than imagined, autonomy would centre trust in principals’ expertise and understanding of their school communities. It would be achieved through a shift towards principal support and development, rather than monitoring and surveillance. Principals would be empowered to meaningfully support young people to learn to live well in a world worth living in (Kemmis et al., 2014). This shift would mean re-thinking notions of impact depending on the context and what would be needed to make educators, students, and communities thrive (Eacott et al., 2021, p. 6). Moving into an increasingly uncertain future, this would be one way of re-professionalising autonomous principals and centring notions of equity, community, and communication in education policy.

## Commentary: the myth of school autonomy and the fantasy of freedom

### Jill Blackmore

Across diverse contexts of Sweden, the United States, Canada, England, Ireland, Australia, Norway and New Zealand there is agreement among educational researchers that school autonomy has fractured, not unified, liberal democracies. Indeed, the social imaginary of autonomy has created myths as to its possibilities which continue to be propagated in policies; for example, parental choice is a right or freedom to choose funded by the state. The neoliberal project has undermined the social contract and reconstituted education, health and welfare, as key sectors of government responsibility have been down-sized, privatised and outsourced. In so doing education, ever more tightly coupled to global capitalism, has been reduced to a private good and not a public good.

The vignettes offered here suggest an emerging realisation (even amongst its former advocates such as the OECD and IMF) that the neoliberal mantra of self-interest, choice and competition, associated with the fantasy of freedom of the autonomous individual based on the binary thinking of the Enlightenment, is a myth (explored by Wilkins, Boyask, Møller, Keddie and O’Neill).

The neoliberal project has proven to have failed to deliver educational equity in democratic societies as the vignettes show.

First, education systems in affluent societies now indicate greater not lesser inequality since school autonomy reforms were introduced in the 1980s, highlighted in Hursh’s vignette of schooling in New York. Second, devolution of governance with school autonomy reform was accompanied by strong external accountabilities in the form of standardised testing and professional standards which, in privileging certain types of knowledge (numerical data), as argued by Skerritt, has denuded education of the core aspects of educating the whole person to be a good citizen as well as worker and individual. These measures have led to epistemic injustice as teacher practitioner knowledge has been devalued as have professional understandings of good practice based on peer and pedagogical relations, as indicated by Stacey et al., Salokangas and McKay. Third, the withdrawal of state provision in education (and health and welfare), coupled with increased regulation and reduced funding, poses dangers for democratic states in times of uncertainty and global crises – wars, pandemics, terrorism, climate disasters, conspiracy theories etc. (see Thomson).

In the context of such uncertainty, there is an imperative and opportunity to reassess school autonomy. Perhaps the focus question should be revised, as Wylie suggests: *What public systems and systems of education are needed to ensure that school autonomy is equitable*?

But, these Anglo-European vignettes illustrate that autonomy is imagined as an ideal in the rhetoric of policy but not experienced as real autonomy by teachers and principals as demonstrated in the contributions of Stacey et al., Salokangas and McKay. Therefore, does autonomy as a concept make sense given that many principals and most teachers feel they are being de-professionalised rather than being valued for their professional judgement. Furthermore, some principals (the focus of policy) enjoy their autonomy and exercise their authority, but often at the expense of teacher autonomy, as argued by Skerritt. Less attention has been paid to teacher autonomy (see Stacey et al.). There is also the risk that external involvement (parents, experts, politicians) in school boards and councils reduces teacher professional autonomy and devalues their expertise.

A related point that these vignettes indicate is a significant tension over the notion of what constitutes the public. Reid (2020, pp. 173–174) argues there are three purposes of education – individual, economic and democratic – for the individual to fulfil their full potential, for work and for engagement in the civil sphere. He also argues that there are three ways of thinking about public education. First, public schools being owned and funded by government with all the obligations involved to educate all students. Therefore, when government funds Academies, free schools or private schools this does not necessarily make them feel obligated to the public. A second version is for the public. The public may comprise a group of individuals with a common interest (e.g. parent and school community as a form of public). In the uneasy tension between public and private good (see Møller), private schools claim they contribute to the public good by teaching social responsibility and civics but do so within a limited version of public restricted to their community (e.g. fund raising – see Yoon). The neoliberal version of the public is the aggregation of individual interests exercising choice.

But the public is also about civil society, the common good arising from shared interests and the public benefits that arise from an inclusive education system which seeks to provide all children with equal opportunity. Reid (2020) offers a third perspective of public education as renewal: the public as proactively seeking a better society. This is about systematically working towards socially just education systems by renewing a sense of the public from a relational perspective of mutuality and sociality.

To change the framing of school autonomy reform we have to move the political blame being placed on failing schools to focus on failing systems and ask: *What would constitute more socially just and enabling systems of school governance and provision?* To do so would require structural and cultural policy shifts for the public good in relation to redistributive justice and the redistribution of resources, recognition of professional knowledge and autonomy, adequate systemic support and mutual accountability between systems and schools:

i. Redistribution of resources based on needs across all sectors/modes of school provision with full financial disclosure for how public funding is distributed.
ii. An accompanying shift in accountability from external standardised assessment ranking to accountability for equity outcomes across all school providers (public and private), i.e. value for the use of public funds.
iii. A shift from external (monitoring and surveillance) to internal forms of accountability which focusses on student and teacher voice in governance and school administration and collegial/peer-based modes of professional accountability more likely to lead to improving student learning.
iv. Refocus principal work onto pedagogy and curriculum and not staffing and budgets, and system provision for additional administrative support in public schools.
v. Top-up of funding for schools which cannot achieve the level of the funds raised by parents in wealthier schools, which would enable schools in disadvantaged communities to offer a wide range of programs beyond welfare, the vocational, literacy and numeracy (e.g. arts, sport programs, etc.).
vi. Provide technical infrastructure, digital software and hardware as well as maintenance costs to enable all students to access resources in a digital world.
vii. Systemic support for recognition and exchange of professional knowledge of teachers and principals.
viii. Reinstate systemic (regional, local) supports and renegotiate the balance of centralisation/decentralisation to benefit schools (e.g. MATs in the UK, the Australian regional and rural schools) and mediate the worst effects of devolved governance.
ix. Focus on inter-agency (health, welfare, government) collaboration to address the socio-geographical distribution of inequality arising from wider social and economic issues that schools cannot resolve but which impacts on their capacity to assist all their students.
x. Feedback mechanisms from schools to central government: mutual responsibility and accountability is more likely to ensure trust in schools and systems with a focus on how systems can enable building professional capacity and student learning.
xi. Local school governance with systemic support via professional development and other supports to recognise diversity in council/board representation and diversity of community. This means first, recognition of the cultural capital of parents as well as experts but disallows politicians on school boards and second, recognition of alternative forms of governance e.g. Indigenous communities led by elders. This requires full financial accountability to avoid corruption.

A revaluing of teaching and education as a public good is central to a democratic society in contemporary and uncertain times. Socially just systems (See O’Neill, 2021; Keddie et al., 2020a, 2020b) require mutuality between the state, schools and individuals based on Fraser’s three principles of social justice: redistribution, recognition and representation.
